# The complete mitochondrial genome of the flea *Hystrichopsylla weida qinlingensis* (Siphonaptera: Hystrichopsylla)

**DOI:** 10.1080/23802359.2022.2053367

**Published:** 2023-04-20

**Authors:** Liangfei Tan, Xuan Yao, Jingyuan Liu, Chaoliang Lei, Qiuying Huang, Bing Hu

**Affiliations:** a College of Plant Science and Technology, Hubei Insect Resources Utilization and Sustainable Pest Management Key Laboratory, Huazhong Agricultural University, Wuhan, China; b Hubei Provincial Center for Disease Control and Prevention, Wuhan, China

**Keywords:** *Hystrichopsylla weida qinlingensis*; *flea*, Hystrichopsylla, mitochondrial genome

## Abstract

The complete mitogenome sequence of the flea, *Hystrichopsylla weida qinlingensis* (Siphonaptera: Hystrichopsylla) was sequenced. The 17,173 bp long genome has the standard metazoan complement of 37 genes. These genes contain 13 protein-coding genes, 22 transfer RNA genes, two ribosomal RNA genes, and one control region. The nucleotide composition of the *H. weida qinlingensis* mitogenome was A: 39.10%, T: 41.49%, G:7.56%, and C: 11.85%. The A + T content is 80.59%, showing strong AT bias. Phylogenetic analysis indicates that Hystrichopsylla has a close affinity with a branch of Dorcadia.

There are more than 2500 species of fleas in the world. They live on the surface of animals, feed on blood, and transmit many diseases (Rust 2005; Bitam et al. 2010; Verhoeve et al. 2020). There are only three families of fleas, and the complete mitochondrial sequences of the four fleas were published, including *Dorcadia ioffi*, *Ctenocephalides felis*, *Jellisonia amadoi*, and *Ceratophyllus wui* (Cameron 2015; Xiang et al. 2017; Tan et al. 2018; Verhoeve et al. 2020).

Hystrichopsyllidae fleas are larger in size, usually with genal comb and pronotal comb, with two rows of bristles on the pronotum, and their female adults have the two spermathecae. There are the two subfamilies under Macropsyllinae and Hystrichopsyllinae, including 55 species in total. In China, there are only 14 species (subspecies) of Hystrichopsyllinae and Hystrichopsylla, which mainly live in the high-altitude mountains and northern regions (*Hystrichopsylla weida qinlingensis* Zhang, Wu *et* Liu, 1984) is an important species of Hystrichopsyllidae, mainly living in the mountain plateau of Central and Western China, including Shennongjia, Enshi and other places in the west of Hubei Province, northwest of Hunan Province, northeast of Chongqing City, southern Qinling of Shaanxi Province, and southwest of Sichuan Province (Kangding). The main host of *H. weida qinlingensis* is *Anourosorex squamipes* (Liu 2018).

Mitochondrial DNA (mtDNA) sequences are essential to species identification and a deeper understanding of evolution of species. The flea *H. weida qinlingensis* belongs to the family of Hystrichopsylla in the order of Siphonaptera which was collected from *Anourosorex squamipes* reported in 2007 (Liu et al. 2007). Here, we elucidated the mtDNA genome of *H. weida qinlingensis*.

The specimens were collected from the Xigou in Hongping Town of Shennongjia (Hubei Province, China) located closely to where *H. weida qinlingensis* were recorded (31°41′ to 31°42′N, 110°24′ to 110°25′E). Now, the specimens are stored in Hubei Provincial Center for Disease Control and Prevention Museum, under the voucher number SNJ1802 (Liangfei Tan: tanliangfei@sina.com). Next-generation sequencing (NGS) technology was used to obtain its complete mitogenome.

The complete mitochondrial genome of *H. weida qinlingensis* is a closed circular molecule 17,173 bp in length and is composed of 37 genes. These genes contain 13 protein-coding genes, 22 transfer RNA genes, two ribosomal RNA genes, and one control region. The nucleotide composition of the *H. weida qinlingensis* mitogenome included A: 39.10%, T: 41.49%, G: 7.56%, and C: 11.85%. The A + T content is 80.59% with obvious AT bias, which was similar with that of the flea *D. ioffi* and *C. felis* (Xiang et al. 2017; Verhoeve et al. 2020). The control region was located between tRNA-Ile and rrnS with a length of 2425 bp, which was the AT-loop region and the A + T content was 90.97%. Four PCGs (ND5, ND4, ND4L, ND1), two rRNAs (rrnL, rrnS), and eight tRNAs (tRNA-Gln, tRNA-Cys, tRNA-Tyr, tRNA-Phe, tRNA-His, tRNA-Pro, tRNA-Leu, and tRNA-Val) were transcribed from the L-strand, while the remaining 23 genes were encoded on the H-strand. ATG was used as the start codon in most genes, namely COX2, ATP6, COX3, ND4, ND4L, and CYTB. ATT was used as the start codon in ND2, ND5, and ND6. ATA was used as the start codon in ND3 and ATP8, and the start codon in COX1 was ATC. Among the 13 PCGs, 12 finally used the TAA stop codon, and only ND3 was used ‘TAG’ as the stop codon.

Molecular Evolutionary Genetics Analysis Version 6.0 (MEGA6.0) was used to construct phylogenetic tree with *H. weida qinlingensis* MtDNA and other blood-sucking insects with maximum-likelihood method (Figure 1) (Tamura et al. 2013). *H. weida qinlingensis* MtDNA was closely clustered with other four previously reported flea species *Jellisonia amadoi*, *D. ioffi*, *C. felis*, and *C. wui*. From the morphological identification, *J. amadoi* and *C. wui* belong to Ceratophyllidae, *H. weida qinlingensis* belongs to Hystrichopsyllidae, *C. felis* belongs to Pulicinae, and *D. ioffi* belongs to Vermipsyllidae. Phylogenetic analysis indicates that the relationship between different species of fleas can be distinguished in the molecular level. The four families are relatively distantly related, and the two species of fleas of Ceratophyllidae are closely related. Hystrichopsylla may have a close affinity with a branch of Dorcadia. Sequencing the complete mitochondrial genome of *H. weida qinglingensis* is helpful to carry out species identification and evolution on the fleas.

**Figure 1.**
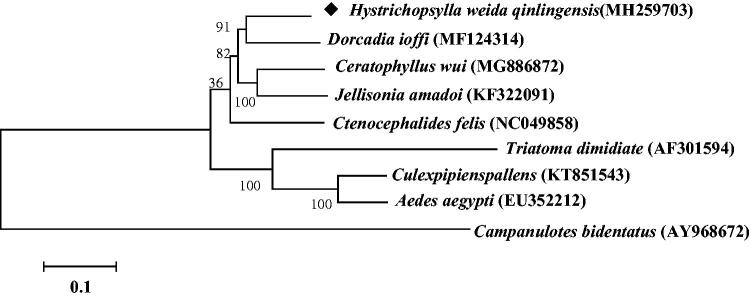
Molecular phylogeny of *Hystrichopsylla weida qinlingensis* and other blood sucking insect species based on the complete mitochondrial genome. The complete mitochondrial genome was downloaded from GenBank and the phylogenic tree was constructed by maximum-likelihood method with 1000 bootstrap replicates.

## Data Availability

The data that support the findings of this study are openly available in the GenBank database at https://www.ncbi.nlm.nih.gov/nuccore/MH259703, under accession number [MH259703]. The associated ‘BioProject’, ‘SRA’, and ‘Bio-Sample’ numbers are PRJNA811436, SRR18186810, and SAMN26332311, respectively.
